# The efficacy of catheter ablation versus ICD for prevention of ventricular tachycardia in patients with ischemic heart disease: a systematic review and meta-analysis

**DOI:** 10.1007/s10840-020-00848-1

**Published:** 2021-03-15

**Authors:** Guolin Liu, Xin Xu, Qijian Yi, Tiewei Lv

**Affiliations:** 1 Department of Cardiology; Ministry of Education Key Laboratory of Child Development and Disorders; National Clinical Research Center for Child Health and Disorders; China International Science and Technology Cooperation base of Child development and Critical Disorders, Children’s Hospital of Chongqing Medical University, Chongqing, 400014 China; 2 Chongqing Key Laboratory of Pediatrics, Chongqing, China

**Keywords:** Implantable cardioverter defibrillator, Ventricular tachycardia, Ischemic heart disease, Catheter ablation

## Abstract

**Purpose:**

Although implantable cardioverter defibrillator (ICD) could prevent the sudden death of ventricular tachycardia (VT) in patients with ischemic heart disease, it could not effectively prevent the recurrence of ventricular tachycardia. Several studies have suggested that catheter ablation may effectively decrease the incidence of ICD events, but relevant dates from randomized controlled trials were limited.

**Methods:**

A systematic review and meta-analysis of randomized controlled trials were performed to evaluate the effect of catheter ablation for the prevention of VT in patients with ischemic heart disease. Random-effects model with inverse-variance weighting method was used to pool odds ratios. Egger method was performed to evaluate whether there was public bias in each outcome.

**Results:**

Four studies enrolling a total of 605 patients were included in the present meta-analysis. Compared with the control group (ICD ± AAD), catheter ablation could significantly reduce the incidence of ICD therapy (OR, 0.49; 95% CI, 0.28 ~ 0.87), ICD shock (OR, 0.50; 95% CI, 0.28 ~ 0.87), VT storm (OR, 0.60; 95% CI, 0.40 ~ 0.90), and cardiovascular-related hospitalization (OR, 0.66; 95% CI, 0.45 ~ 0.9). But there was no significant difference among the risk of all-cause mortality (OR, 0.89; 95% CI, 0.59 ~ 1.34), cardiovascular mortality (OR, 0.76; 95% CI, 0.44 ~ 1.30), and complication (OR, 0.89; 95% CI, 0.30 ~ 2.67).

**Conclusion:**

These results showed that catheter ablation combined with ICD could reduce ICD therapy, ICD shock, and VT storm in patients with ischemic heart disease, but there was no improvement in all-cause mortality. Meanwhile, it also provided a basic guidance for the design of larger clinical randomized trials with longer follow-up in the future.

**Electronic supplementary material:**

The online version of this article (10.1007/s10840-020-00848-1) contains supplementary material, which is available to authorized users.

## Introduction

Ventricular tachycardia (VT) is the main cause of sudden death in patients with ischemic heart disease (IHD). Implantable cardioverter defibrillator (ICD) has been proven to reduce the risk of sudden cardiac death by anti-tachycardia pacing (ATP) and ICD shock so that it has become the most effective measure for the primary or secondary prevention of sudden cardiac death [1]. But several studies have shown that ICD shock, whether appropriate or inappropriate, could increase mortality [2–4]. At the same time, frequent ICD shock also could cause clinical post-traumatic stress disorder and reduce the quality of life of patients [5–7].

Although optimized device programming might reduce the risk of ICD shock in all patients with ischemic heart disease, it could not effectively prevent the recurrence of VT [8–10]. In addition, the combination of antiarrhythmic drug (AAD) with ICD could also reduce the recurrence of VT and frequency of ICD therapy, but many patients are unable to take medicine continuously because of the poor tolerance caused by adverse side reactions [11, 12].

Since catheter ablation (CA) has been applied to the treatment of arrhythmias in patients with IHD, several clinical randomized controlled trials (RCTs) have been carried out and the outcomes suggested that catheter ablation might prevent the recurrence of ventricular tachycardia, but the results were still controversial owing to the limited relevant data [13]. Therefore, the aim of the present meta-analysis of randomized controlled trials was to further confirm that whether catheter ablation could reduce ICD therapy, ICD shock, VT storm, and mortality in patients with ischemic heart disease and conduct an assessment of its efficacy and safety.

## Method

We followed the PRISMA (Preferred Reporting Items for Systematic Reviews and Meta-Analyses) guidelines for all stages of design and implementation throughout the process [14].

### Search strategy

The literature search was carried out on PubMed, Embase, and Cochrane Library, without language restrictions. The keywords we used were “ventricular tachycardia,” “ischemic heart disease” or “Coronary Artery Disease,” and “catheter ablation” and were limited by human trials and randomized controlled trials. In addition, we reviewed the relevant reviews to look for the potential missing studies and obtained the scripts of studies that were not retrieved initially.

### Selection criteria

The literature inclusion criteria were as follows: (1) randomized controlled trial; (2) patients enrolled ≥ 18 years old; (3) patients with ischemic heart disease have been implanted or were ready to be implanted with ICD; (4) the study group was CA + ICD, control group was ICD (with or without AAD); (5) the sample size was more than 50; and (6) the study reported at least 3 items of following endpoints—ICD therapy (ATP + ICD shock), ICD shock, VT storm, all-cause mortality, cardiovascular mortality, cardiovascular-related hospitalization, and complication.

Exclusion criteria: non-randomized controlled trials, animal trials, and studies enrolled patients with non-ischemic heart disease.

### Data extraction and outcome measurement

The data extraction was performed independently by two researchers (X.X and Q.J.Y). The baseline characteristics of patients included age, male, size, time from myocardial infarction (MI) to enrollment, left ventricular ejection fraction (LVEF), hypertension (HTN), and diabetes mellitus (DM) (Table 1). The characteristics of the included study included year, sample size, comparator, procedure design, inclusion criteria, epicardial ablation, amiodarone and beta-blocker, follow-up, cross-over to ablation, and complication related to ablation (Table 2).

**Table 1:** Baseline characteristics of patients

Study	Age	Male (%)	Size	Time from MI to enrollment (years)	LVEF (%)	HTN (%)	DM (%)
SMASH-VT	67/66	92/81	64/64	Ablation: 8.8 ± 8.5	Ablation: 30.7 ± 9.5	73/67	38/50
				Control: 7.9 ± 7.8	Control: 32.9 ± 8.5		
VTACH	68/64	96/91	52/55	Ablation: 12.6 ± 8	Ablation: 34 ± 9.6	NR	NR
				Control: 13.3 ± 8.6	Control: 34.1 ± 8.8		
VANISH	68/70	93/93	132/127	NR	Ablation: 31.1 ± 10.4	70/69	28/30
					Control: 31.2 ± 10.7		
SMS	68/66	87/81	54/57	Ablation: 11.1 ± 6.6	Ablation: 32 ± 6.9	NR	NR
				Control: 8.6 ± 7.8	Control: 30.4 ± 7.3		

NR = not reported; HTN = hypertension; DM = diabetes mellitus; MI = myocardial infarction; LVEF = left ventricular ejection fraction

**Table 2:** Study characteristics

Study	SMASH-VT	VTACH	VANISH	SMS
Year	2007	2010	2016	2017
Sample size	128	107	259	111
Comparator	ICD alone	ICD alone	ICD + escalating AADs	ICD alone
Procedure design	Substrate modification in sinus rhythm	Ablation in stable VT, and substrate modification in case of non-inducible or unstable VT.	Standardized approach targeted all inducible VT.	Substrate modification
Included criteria	MI > 1 month; planned or recent (within 6 months) ICD for VF, unstable VF, or syncope with inducible VF	Indication for an ICD as secondary prevention for documented stable clinical VT without any reversible cause, CAD, MI, LVEF ≤ 50%	Myocardial infarction, an ICD, episode of ventricular tachycardia when using class I or class III AAD within the previous 6 months	CAD, LVEF ≤ 40%, unstable spontaneous VT, cardiac arrest or syncope with unstable VT inducible
Epicardial ablation	NR late potential	NR	NR	NR
Amiodarone	0	35% ablation 35% control	66.1% ablation 64.2% control	30% ablation 35% control
BB (%)	94/98	75/75	93.9/96.1	91/91
Follow-up (months)	22.5 ± 5.5	22.5 ± 9	27.9 ± 17.1	27.6 ± 13.2
Cross-over to ablation	NR	12	11	1
Complication related to ablation	Pericardial effusion without tamponade (1), exacerbation of congestive heart failure (1), deep venous thrombosis (1)	None	Vascular injury (3), cardiac perforation (2), heart block (1)	Third-degree atrioventricular conduction block (2), tamponade requiring pericardiocentesis (2)

ICD = implantable cardioverter defibrillator; BB = beta-blockers; AAD = antiarrhythmic drug; VT = ventricular tachycardia; VF = ventricular fibrillation; CAD = coronary artery disease; MI = myocardial infarction; IHD = ischemic heart disease; NR = not reported

The primary outcomes of interest were ICD therapy (ATP + ICD shock), ICD shock, and VT storms. Additional outcomes were all-cause mortality, cardiovascular mortality, cardiovascular-related hospitalization, and complication. VT storm was defined as more than three ICD shocks within 24 h, and ICD therapy was defined as ICD shock and anti-tachycardia pacing (ATP). Any differences will be approved with the third independent researcher (G.L.L) and all the problems were solved after discussion.

### Quality assessment

The Cochrane risk of bias (ROB) tool was used for RCTs for six dominants: random sequence generation, allocation concealment, blinding of participants and personnel, incomplete outcome data, selective reporting, and other bias. The evaluation results are divided into low risk, unclear, and high risk [15]. Almost studies were at low or unclear risk in selection bias, detection bias, attrition bias, and reporting bias, but owing that the included RCTs were to compare the effect of catheter ablation on the prevention of ventricular tachycardia in patients with IHD, it was too difficult to perform blind method, which might lead to a high risk of performance bias among the studies. The details are described in Table 3.

**Table 3:** Risk-of-bias assessment of included randomized controlled trials

Citation: name and year	Random sequence generation	Allocation Concealment	Blinding of participants	Blinding of outcome assessment	Incomplete outcomes data	Selective reporting	Other bias	Risk of bias
SMASH-VT 2007	Unclear	Low	High	Unclear	Low	Low	Low	Low
VTACH 2010	Low	Low	High	Unclear	Unclear	Low	Unclear	Moderate
VANISH 2016	Low	Low	High	Unclear	Low	Low	Unclear	Low
SMS 2017	Low	Unclear	High	Unclear	Low	Low	Low	Low

Risk of bias was assessed with use of the Cochrane risk of bias tool. The overall risk of bias of a study was considered “low” if > 4 items were rated as “low risk” and “moderate” if 2 or 3 items were rated as “low risk.” The overall risk of bias of a study was considered “high” if < 2 items were rated as “low risk” or if > 1 item was rated as “high risk”

### Statistical analysis

Statistical analysis was performed with the STATA MP15 (Stata Corp, LLC). Binary variables were reported as odds ratio (OR) with 95% confidence intervals (CIs), which were pooled by random-effects model with inverse-variance weighting, and *P* < 0.05 was considered to be statistically significant difference [15]. The statistical heterogeneity among studies was evaluated by I-square statistics and I^2^ < 50% was considered as no significant heterogeneity and I^2^ ≥ 50% was considered as significant heterogeneity. Egger method was used to evaluate the publication bias.

## Results

### Extraction of articles

**Fig. 1:**
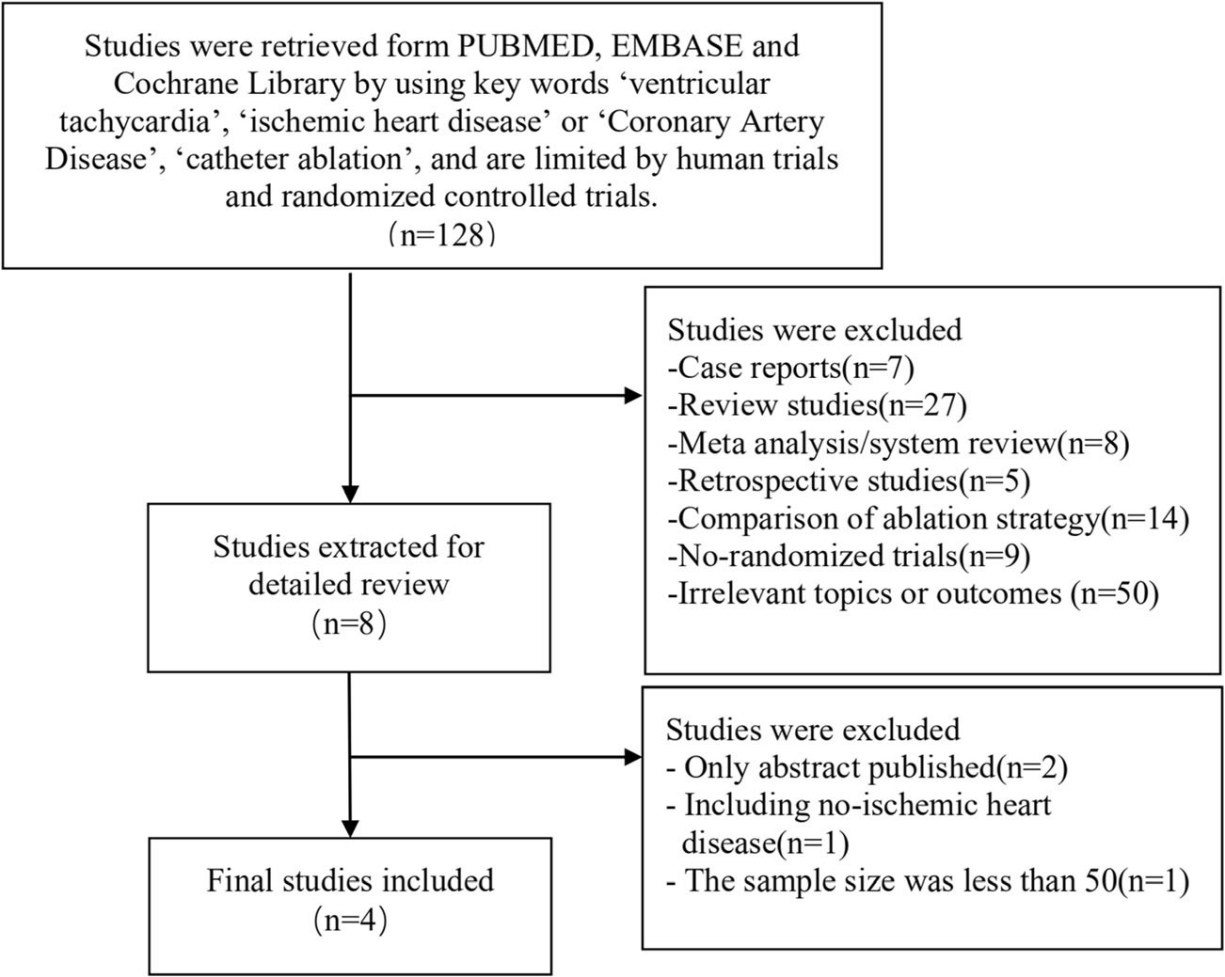
The selection flowchart of literature screening for the meta-analysis

We searched all the studies as of January 2020 in PubMed, Embase, and Cochrane Library and found a total of 128 articles, no eligible articles from other sources. Then, 120 articles were excluded according to the title and abstract, and the remaining 8 articles were browsed full text. However, 2 articles had only abstract and no specific outcomes, 1 article included patients with non-ischemic heart disease, and the sample size of 1 article without major results was less than 50. Finally, 4 randomized controlled trials were included in the present meta-analysis [16–19]. The detailed screening process is shown in Fig. 1.

### Study characteristics

A total of 605 patients were included in the four studies, who were intention-to-treat (ITT), with a mean age of 64 to 70 years old, including 90.1% of male, and the follow-up period ranged from 6 to 27.9 months.

In SMASH-VT, about 87% of patients had ICD implanted before ablation [18]. In VTACH, patients in the ablation group were implanted with ICD after ablation [16]. However, in SMS, patients in the ablation group were implanted with ICD before ablation [17]. Surprisingly, all patients in VANISH had received ICD therapy at the time of registration [19].

The use of class I or III antiarrhythmic drug at baseline and during follow-up was also significantly different in the patients enrolled in each study. In SMASH-VT, patients have not used class I or III antiarrhythmic drugs at baseline until they reached the primary endpoint [18]. In VTACH, the utilization rate of amiodarone in the two groups at baseline was 35%, and the rates of amiodarone in the ablation group and the control group were 26% (12/46) and 31% (15/48), respectively, at 12 months [16]. In VANISH, 65.3% of patients took amiodarone at baseline (169/259) [19]. Additionally, 32% of SMS took amiodarone at baseline, while the rates of amiodarone in the ablation group and the control group were 31% (9/29) and 27% (8/30), respectively, at 3 years [17].

### Clinical outcomes

**Fig. 2:**
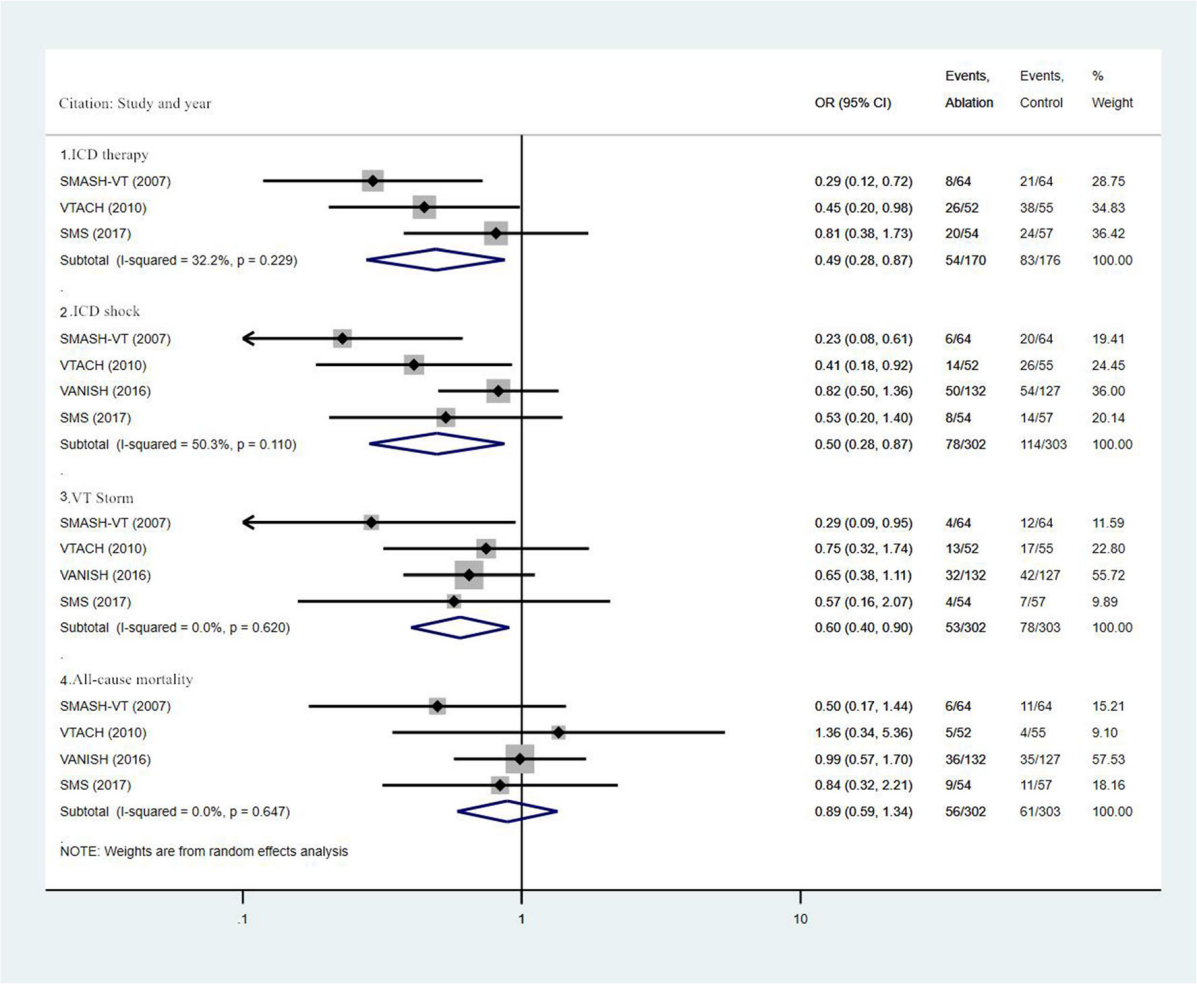
Summary forest plot of ICD therapy, ICD shock, VT storm, and all-cause mortality

**Fig. 3:**
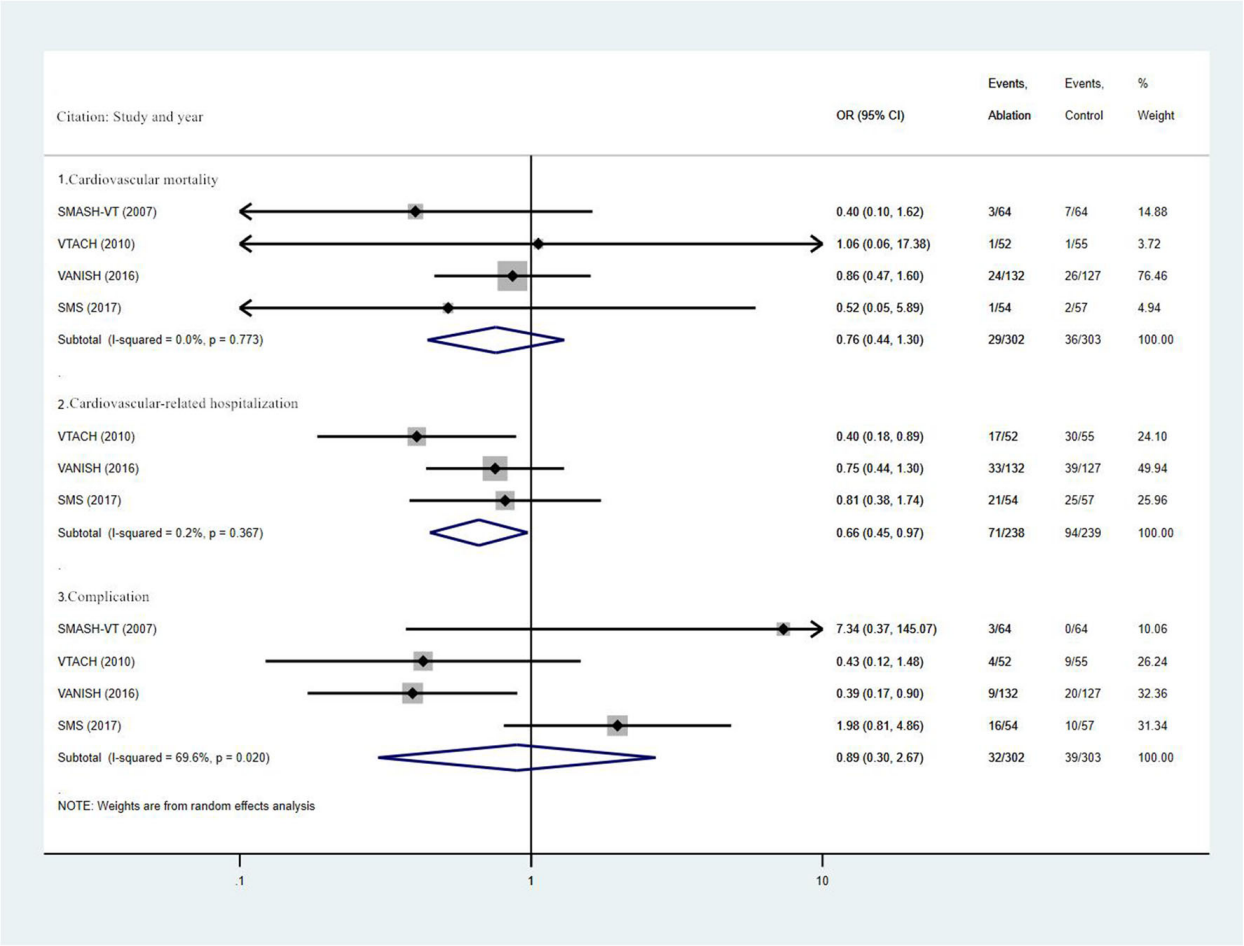
Summary forest plot of cardiovascular mortality, cardiovascular-related hospitalization, and complication

The weighted OR values of each outcomes between the two groups are shown in Fig. 2 and Fig. 3. Compared with the control group, catheter ablation combined with ICD could reduce the incidence of ICD therapy (OR, 0.49; 95% CI, 0.28 ~ 0.87; I^2^ = 32.2%; *P* = 0.229), ICD shock (OR, 0.50; 95% CI, 0.28 ~ 0.87; I^2^ = 50.3%; *P* = 0.110), VT storm (OR, 0.60, 95% CI, 0.40 ~ 0.90, I^2^ = 0%; *P* = 0.620), and cardiovascular-related hospitalization (OR, 0.66; 95% CI, 0.45 ~ 0.97; I^2^ = 0.2%; *P* = 0.367), and there was statistically significant statistical difference. Moreover, although there was a downward trend that the combined use of catheter ablation with ICD was able to reduce the risk of all-cause mortality (OR, 0.89; 95% CI, 0.59 ~ 1.34; I^2^ = 0.0%; *P* = 0.647), cardiovascular mortality (OR, 0.76; 95% CI, 0.44 ~ 1.30; I^2^ = 0.0%; *P* = 0.773), and complication(OR, 0.89; 95% CI, 0.30 ~ 2.67; I^2^ = 69.6%; *P* = 0.020), there was no statistical difference ***(***Figs. 2 and 3***)***.

### Public bias assessment and evidence quality level

The GRADE assessment tool was used to evaluate the recommended levels of each outcomes. The evidence quality level of ICD therapy and complications was moderate, and that of ICD shock, VT storm, all-cause mortality, cardiovascular mortality, cardiovascular-related hospitalization, and complication were high. Moreover, there was no significant publication bias in all outcomes (*P* = 0.300, 0.120, 0.427, 0.769, 0.531, 0.638, and 0.609 for ICD therapy, ICD shock, VT storm, all-cause mortality, cardiovascular mortality, cardiovascular-related hospitalization, and complication, respectively) (Supplement materials).

### Sensitive analysis

**Fig. 4:**
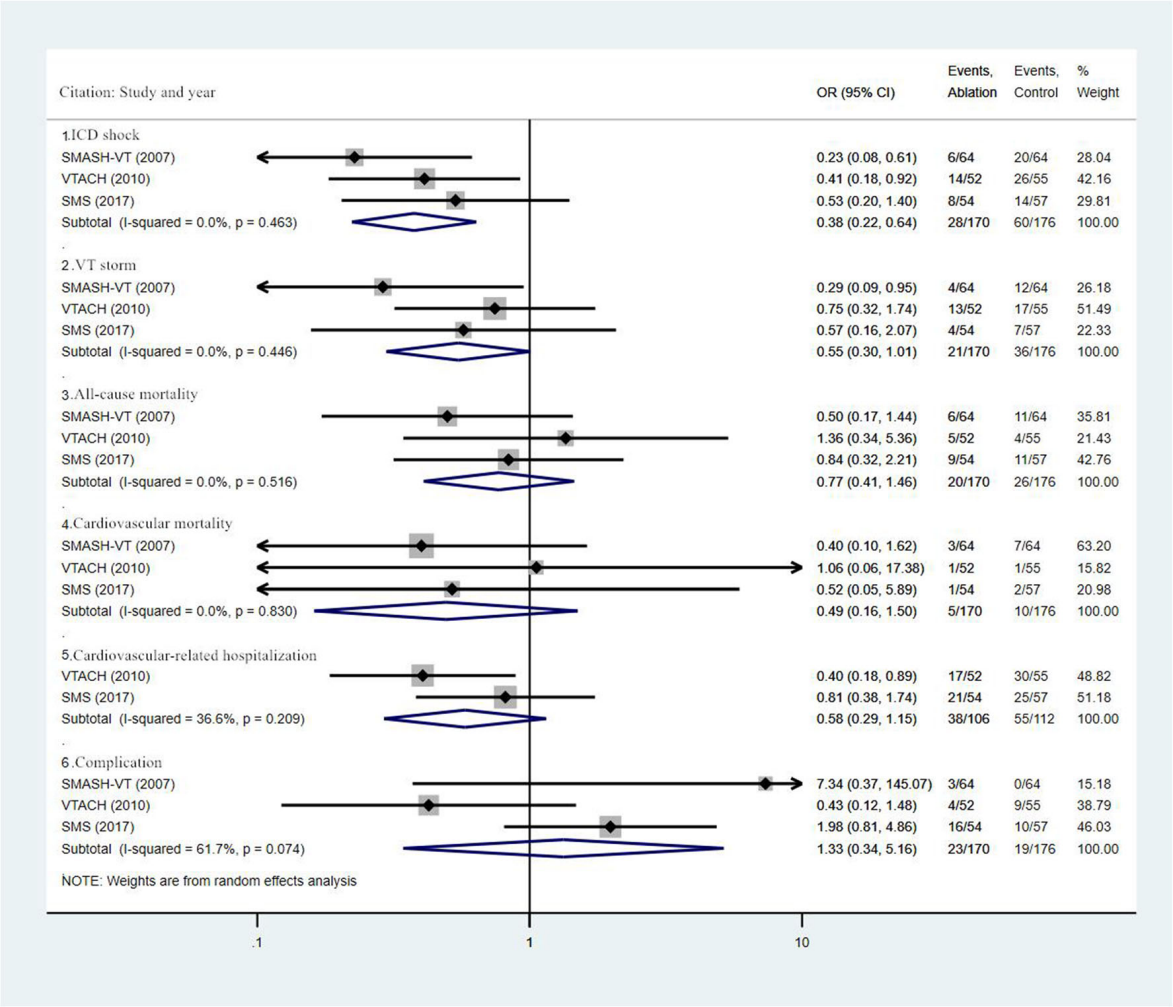
Summary forest plot for sensitive analysis of ICD shock, VT storm, all-cause mortality, cardiovascular mortality, cardiovascular-related hospitalization, and complication

The control group in the VANISH study was patients treated with ICD + ADD, which might result in a significant risk of other bias [19]. Therefore, we carried out the sensitive analysis through excluding this study and the results are shown in Fig. 4. We found that the incidence of ICD shocks in the ablation group further decreased obviously (OR, 0.38, 95% CI, 0.22 ~ 0.64, I^2^ = 0.0%; *P* = 0.463), which further confirmed the effectiveness of CA in the prevention of VT in patients with IHD. Interestingly, the incidence of VT storm (OR, 0.55, 95% CI, 0.30 ~ 1.01, I^2^ = 0.0%; *P* = 0.446), all-cause mortality (OR, 0.77, 95% CI, 0.41 ~ 1.46, I^2^ = 0.0%; *P* = 0.516), cardiovascular mortality (OR, 0.49, 95% CI, 0.16 ~ 1.50, I^2^ = 0.0%; *P* = 0.830), and cardiovascular-related hospitalization (OR, 0.58, 95% CI, 0.29 ~ 1.15, I^2^ = 36.6%; *P* = 0.209) also decreased.

## Discussion

Ventricular tachycardia in patients with ischemic heart disease is mainly caused by the reentrant mechanism of scar myocardium, and it is a potentially fatal rapid arrhythmia and increases the risk of sudden cardiac death [20, 21]. Sudden cardiac death is currently the most difficult to predict cardiovascular events in the cardiovascular field. Due to the vast majority of sudden cardiac death occurring outside the hospital, it is difficult to get timely and effective treatment, resulting in a high mortality rate. Although the current guidelines suggest ICD implantation to improve the prognosis of patients with ventricular arrhythmias as a class I recommendation, frequent ICD shocks could not only reduce the quality of life and cardiac function of patients but also lead to post-traumatic anxiety and depression and increase the risk of death [22]. In addition, multiple discharges of ICD could result in overuse of electricity and reduce the life of ICD. Therefore, how to reduce the frequency of ICD shock, improve the quality of life, and reduce mortality has been the focus of clinicians.

Although the results of present meta-analysis suggest that catheter ablation could not significantly reduce the risk of all-cause mortality and cardiovascular mortality, it has a downward trend. On the contrary, it also indirectly suggests that catheter ablation does not increase the risk of death in patients with ICD. Moreover, although patients had a risk of suffering from ablation-related complications, mainly pericardial effusion, there were no deaths during ablation in all studies. Meanwhile, SMASH-VT suggested that ablation had little effect on cardiac function, which also confirmed the safety of catheter ablation in patients with ischemic heart disease [18].

VTACH and SMS both showed that ablation therapy could prolong the time to first recurrence of VT/VF, and the former showed significant difference, but the latter did not, which may be due to the differences in their respective criteria for enrollment of patients [17, 19]. Patients with stable ventricular tachycardia were mainly included in VTACH, while patients with unstable ventricular tachycardia were mainly included in SMS. In addition, the VTACH study also showed that catheter ablation can improve the survival rate of freedom from ventricular tachycardia in patients with LVEF > 30% (HR, 10.47; 95% CI, 0.24 ~ 0.88), but there was no significant difference in patients with LVEF ≤ 30% between the two groups [16]. These results suggest that catheter ablation may decrease the recurrence rate of ventricular arrhythmias in patients with LVEF > 30% but may not be effective in patients with LVEF ≤ 30%. Moreover, in the MADIT trial, ICD reduced the relative risk of death by 54% [23]; the MADIT-II trial proved that ICD can effectively reduce the total mortality of patients with cardiac insufficiency (EF ≤ 30%) after myocardial infarction [24] and the MUSTT trial proved that ICD treatment can significantly reduce arrhythmic death or cardiac arrest and total mortality [25]. However, it is impossible for us to carry out further subgroup analysis due to the enrollment criteria difference of the inclusion studies. Therefore, larger randomized controlled trials with longer follow-up period were needed to further explore the effects of catheter ablation on the recurrence of VT, quality of life, and mortality in patients with ischemic heart disease.

Notably, our meta-analysis did not distinguish the ablative timing of the patients enrolled and the optimum chance for catheter ablation in patients with ischemic heart disease is still unclear. Fortunately, BERLIN-VT, a recent randomized controlled trial, has compared the effects of prophylactic and delayed ablation on the prevention of ventricular tachycardia in patients with ischemic heart disease and the results showed that there was no significant difference in all-cause mortality and cardiovascular-related hospitalization rates between the two groups, but the prophylactic ablation group significantly shortened the time of first hospitalization due to the deterioration of heart failure and higher incidence of death of other cause [26]. Interestingly, the sustained VT/VF recurrence (39.7% vs 48.2%) and appropriate ICD therapy (34.2% vs 47.0%, *P* = 0.030) significantly decreased in the prophylactic ablation group [26]. Nevertheless, prophylactic catheter ablation is still infeasible to be recommended as the first choice owing that not each patient with ischemic cardiomyopathy will have a possibility to develop ventricular tachycardia. Therefore, catheter ablation should be delayed until recurrence of ventricular tachycardia is documented after ICD implantation in almost patients with ischemic cardiomyopathy and at risk of sudden cardiac death, which also is exactly what the guidelines recommend [27].

Catheter ablation of the pathological matrix of patients with ischemic cardiomyopathy can effectively reduce ICD events and VT recurrence, which can be more effective and safer for such patients with the extensive application of the three-dimensional mapping system. A large retrospective study showed that among 2061 structural heart disease patients, 87% of whom had been implanted with ICD, catheter ablation can reduce 70% of VT recurrences and the absence of VT recurrence had a significant relationship with the reduction of all-cause mortality [28]. Although our meta-analysis also showed that catheter ablation may significantly reduce VT storm, it could not significantly reduce all-cause mortality as mentioned above, which may be accounted for the difference of cardiac function and ejection fraction of the patients enrolled in different research. Therefore, larger and more targeted randomized controlled trials are needed to further confirm relevant conclusions.

## Limitation

This meta-analysis has several limitations. Firstly, only 4 randomized controlled trials and a small number of patients were included so that subgroup analysis could not be carried out, which has a certain impact on the reliability of the outcomes. Secondly, although there were no significant heterogeneity and publication bias in each outcomes of meta-analysis, all the included studies not using blind methods could lead to performance bias. Thirdly, the enrollment criteria of the patients in our study were not consistent. For example, the patients of control group in the VANISH study were treated with antiarrhythmic drugs during the whole follow-up, but similar patients were excluded in the VTACH study, which may be one of the main reasons for the heterogeneity. Fourthly, inconsistent success rates of catheter ablation owing to different ablation strategies and different follow-up time both could have a certain impact on the rate of VT recurrence. Finally, the comparison for quality assessment of life between the two groups, which was often a quite important indicator of clinical efficacy, was not conducted in the included studies so that the present meta-analysis could not further summarize and analyze.

## Conclusion

In summary, for ventricular arrhythmias in patients with ischemic heart disease, combined use of ICD with catheter ablation could significantly reduce the incidence of ICD therapy, ICD shock, VT storm, and cardiovascular-related hospitalization, which also suggested that catheter ablation may become an effective clinical treatment option, but it could not significantly reduce the risk of all-cause mortality, cardiovascular mortality, and complication.

## Electronic supplementary material

ESM 1 (RAR 667 kb)
